# Biopsy-proven acute eosinophilic myocarditis as the initial manifestation of severe primary Sjögren's syndrome: a case report

**DOI:** 10.3389/fcvm.2025.1683444

**Published:** 2025-10-08

**Authors:** Katsuya Hashimoto, Hiroyuki Yamamoto, Jun Isogai, Yoshihiko Ikeda, Masaaki Hamada, Yuichi Dai, Toru Hashimoto

**Affiliations:** ^1^ Department of Cardiovascular Medicine, Narita-Tomisato Tokushukai Hospital, Chiba, Japan; ^2^ Department of Cardiology, Tokyo Medical University Hospital, Tokyo, Japan; ^3^ Department of Radiology, Asahi General Hospital, Asahi, Japan; ^4^ Department of Pathology, National Cerebral and Cardiovascular Center, Suita, Japan; ^5^ Department of Endoscopy, Narita-Tomisato Tokushukai Hospital, Chiba, Japan; ^6^ Diagnostic Pathology, Tsukuba Memorial Hospital, Ibaraki, Japan

**Keywords:** acute eosinophilic myocarditis, primary Sjögren's syndrome, acute otitis media, digestive involvement, ulcerative colitis

## Abstract

**Background:**

Primary Sjögren's syndrome (pSS) is a chronic autoimmune inflammatory disorder primarily affecting the exocrine glands. A subset of patients exhibits extraglandular manifestations, including cardiovascular involvement. Among them, myocarditis is a rare complication, and its pathogenesis remains poorly understood.

**Case presentation:**

An 85-year-old man with a persistent dry mouth was admitted to our hospital with high-grade fever, nausea, fatigue, and urinary disturbance. On day 2, the patient developed multiple cerebral infarctions and bilateral acute otitis media. Fever and inflammatory response without leukocytosis and cardiac imaging findings indicative of active myocarditis, and normal cardiac function suggested acute viral myocarditis, for which supportive treatments were initiated. On day 6, the patient experienced acute heart failure with severely reduced ejection fraction and cardiogenic shock. An endomyocardial biopsy was performed following transient peripheral eosinophilia in serial blood samples, which revealed acute eosinophilic myocarditis (AEM). A thorough diagnostic evaluation for eosinophilia revealed pSS, leading to the final diagnosis of pSS-associated AEM. Systemic high-dose corticosteroid treatment improved the general condition of the patient, except for a left ventricular apical aneurysm. Nevertheless, the patient's post-treatment hospital course was complicated by serious digestive involvement, leading to death from septic shock.

**Conclusions:**

To our knowledge, this is the first case of severe pSS complicated by AEM. This case highlights the importance of early therapeutic intervention for AEM and early comprehensive surveillance of systemic organs for pSS. Furthermore, this case provides new insights into the pathogenesis of pSS-associated myocarditis.

## 1 Introduction

Sjögren's syndrome (SS) is a multisystemic autoimmune inflammatory disorder characterized by lymphocytic infiltration of the lacrimal and salivary glands. SS commonly affects middle-aged individuals, with a strong female predominance (1). Primary Sjögren's syndrome (pSS) presents with sicca symptoms, but extraglandular involvement can occur in a multiorgan system (2). Although cardiovascular involvement may lead to pericarditis and heart block, myocarditis is rare, and its pathogenesis remains poorly understood (3).

## 2 Case description

**Figure 1:**
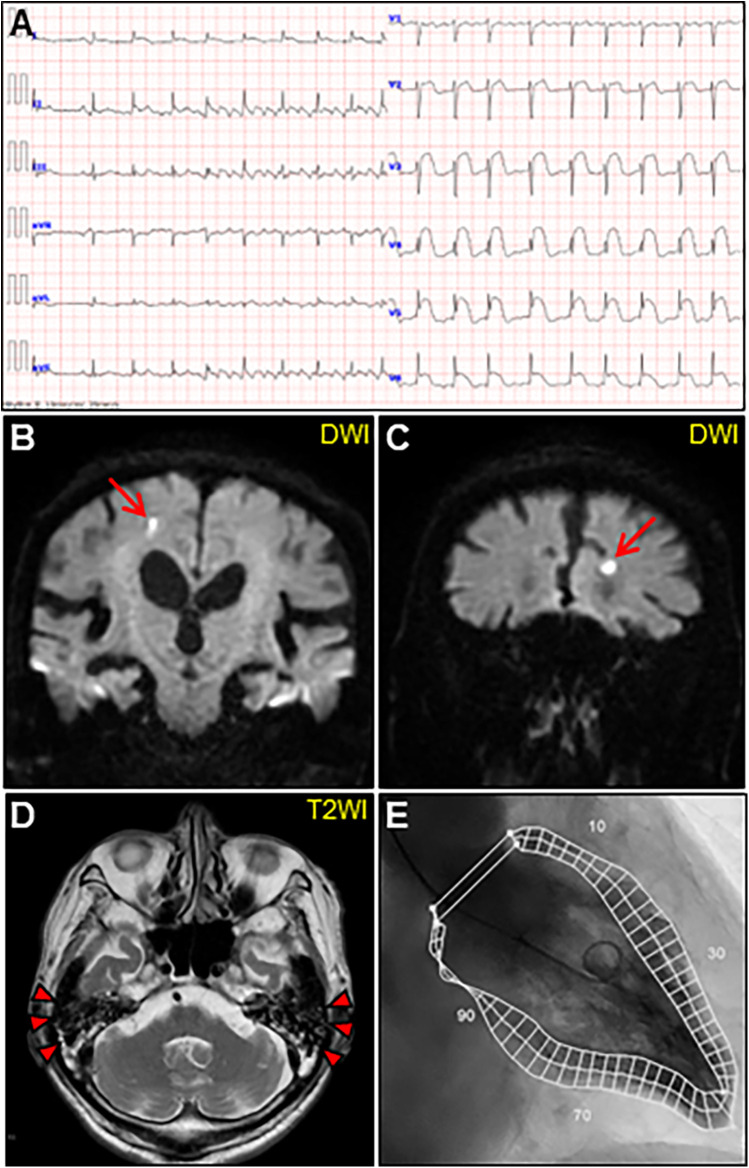
Initial examinations. **(A)** Initial electrocardiography. (B, C) Coronal views of brain diffusion-weighted magnetic resonance images (DWIs) showing multiple acute cerebral infarctions (arrows). **(D)** T2-weighted image (T2WI) showing fluid collection as a hyperintense area in bilateral mastoid cavities (arrowheads). **(E)** Left ventriculogram on day 2 after admission showing normal hyperkinetic contractions with an ejection fraction of 60%.

**Figure 2:**
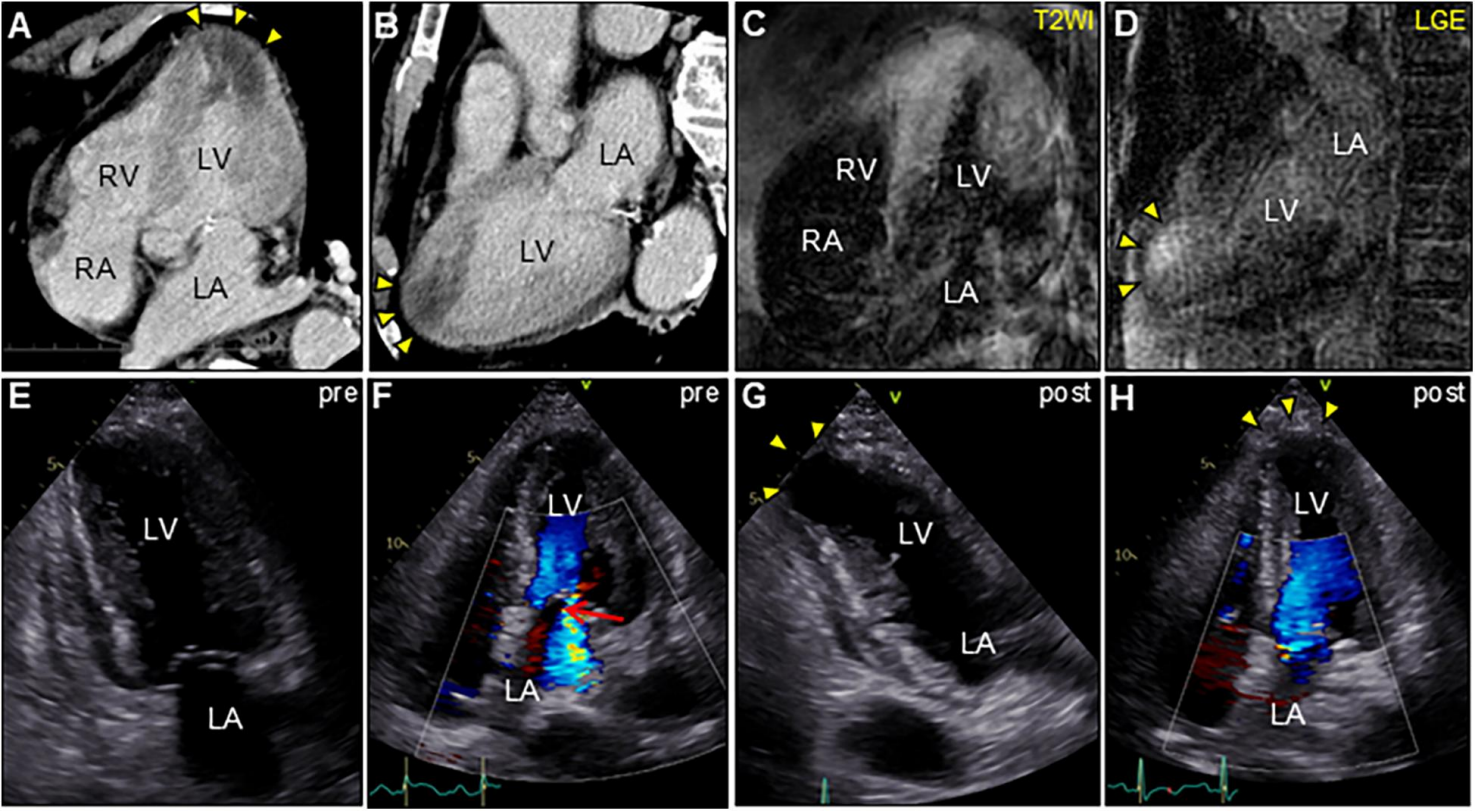
Enhanced computed tomography (CT) and cardiac magnetic resonance imaging (CMR) (**A,C**, apical four-chamber view; **B,D**, long-axis view). CT showing low-attenuation areas of the left ventricular apex (arrowheads). T2-weighted image (T2WI) showing diffuse myocardial edema. Late gadolinium enhancement (LGE) images showing the same region as that of CT (arrowheads). Serial transthoracic echocardiography (TTE). (**E,G**, apical two-chamber view; and **F,H**, apical four-chamber view). **(E,F)** TTE on day 6 after admission showing severe global left ventricular systolic dysfunction with increased left ventricular wall thickness (LVWT) accompanied by severe mitral regurgitation (arrow) in color Doppler mode [LVWT, 14 mm; and ejection fraction (EF), 40%]. **(G,H)** Follow-up TTE 2 weeks after corticosteroid treatment showing considerable improvement in left ventricular function and mitral regurgitation (LVWT, 12 mm; and EF, 57%). Note the left ventricular apical aneurysmal formation (arrowheads). LA, left atrium; LV, left ventricle; RA, right atrium; RV, right ventricle.

**Figure 3:**
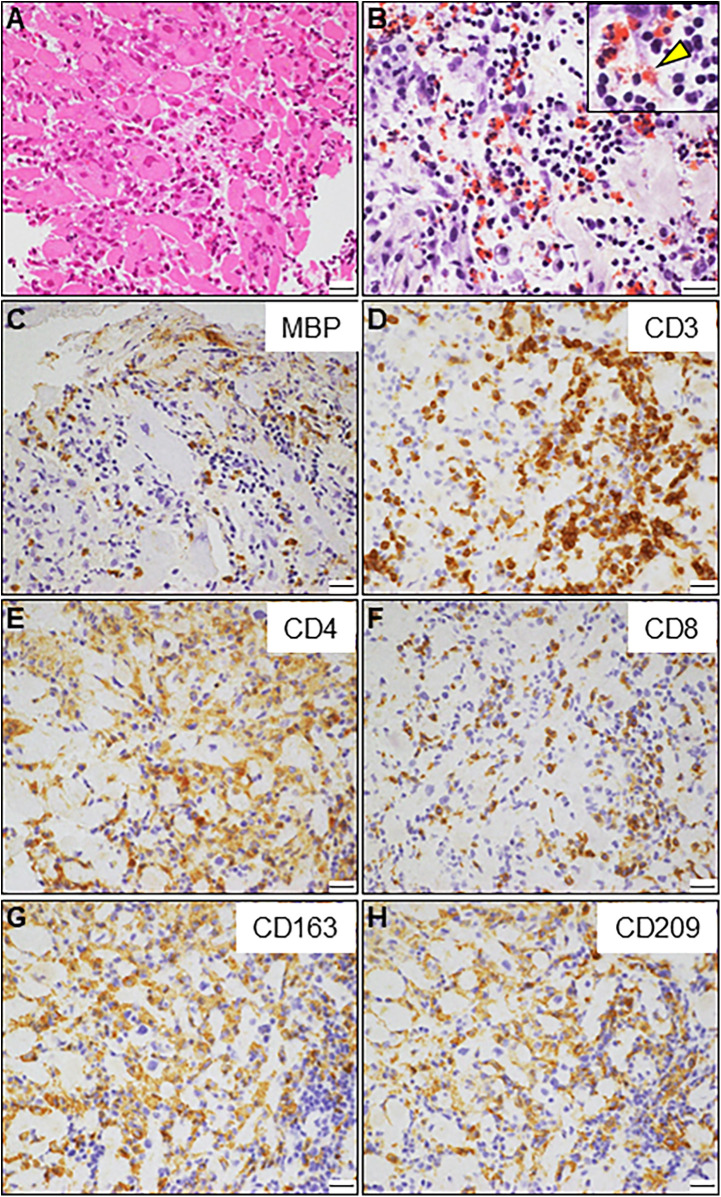
Endomyocardial biopsy. **(A)** Hematoxylin and eosin staining. **(B)** Direct fast scarlet staining showing intense eosinophilic infiltration. The inlet shows a magnified view of degranulated eosinophilic granules (arrowhead). **(C)** Immunostaining for major basic proteins (MBP). (**D–F**) Immunohistochemistry showing abundant inflammatory infiltrates comprising CD3 + and CD4+ T-lymphocytes. A small number of CD8+ T lymphocytes are also observed. (G, H) A considerable number of CD163 + and CD209+ M2 macrophages are observed. Scale bars: 20 μm.

An 85-year-old man with high-grade fever, nausea, fatigue, and urinary disturbance was admitted to our hospital. The patient had a history of hypertension and benign prostatic hyperplasia, but no history of autoimmune gastrointestinal disorders. His vital signs were as follows: blood pressure, 160/102 mmHg; heart rate, 99 beats/min; and body temperature, 38.2°C. A physical examination revealed a distended abdomen. Abdominal computed tomography (CT) revealed an enlarged prostate gland with a distended bladder wall and bilateral hydronephrosis, suggesting a urinary obstruction. Empirical antimicrobial treatment with intravenous ceftriaxone was initiated for suspected urinary tract infection. On day 2, the patient became agitated, disoriented, and unable to follow instructions. Neurological examination revealed left hemiparesis and profound bilateral hearing loss. Electrocardiography showed atrial fibrillation with tachycardia and ST-segment elevation in the precordial leads (Figure 1A). Brain magnetic resonance imaging (MRI) revealed multiple cerebral emboli and bilateral acute otitis media (AOM) (Figures 1B–D). Laboratory tests revealed a white blood cell count of 6,200/*μ*l without eosinophilia and an elevated C-reactive protein (CRP) level of 7.47 mg/dl (normal: <0.3 mg/dl). Additionally, elevated levels of serum cardiac troponin I (cTnI; 11,023 pg/ml, normal: <26.2 pg/ml) and N-terminal pro-brain natriuretic peptide (NT-proBNP; 885 pg/ml, normal: <125 pg/ml) were observed. Coronary angiography and left ventriculography were unremarkable (Figure 1E). Echocardiography revealed left ventricular (LV) hypertrophy, preserved LV contraction, and mild pericardial effusion. Enhanced CT indicated acute myocardial edema of the LV apex involving the anterolateral pupillary muscle (Figures 2A,B). Cardiac MRI established acute myocarditis (Figures 2C,D). The repeated urine and blood cultures remained sterile. The fever and inflammatory response without leukocytosis raised the suspicion of acute viral myocarditis with concomitant cardiogenic emboli. The patient underwent close telemetric monitoring with intravenous heparin infusion, followed by oral anticoagulant and anti-heart failure treatment. Furthermore, acetaminophen was administered for AOM. On day 6, the patient was transferred to the intensive care unit (ICU) due to cardiogenic shock, requiring intubation and catecholamine support. Follow-up electrocardiography revealed pathological Q waves with complete ST-segment resolution. Follow-up echocardiography revealed severe diffuse global hypokinesis with severe mitral regurgitation (Figures 2E,F; Supplementary Videos S1 and S2). Follow-up laboratory examinations revealed a greater increase in serum cTnI (18,946 pg/ml), NT-proBNP (35,000 pg/ml), and CRP (10.08 mg/dl) levels. Note the transient increase in the circulating absolute eosinophilic count to 542 cells/μl (Supplementary Figure S1). Repeated left ventriculography confirmed reduced global systolic function with an ejection fraction of 35%, followed by the insertion of an intra-aortic balloon pump (IABP). Subsequent endomyocardial biopsy (EMB) revealed numerous eosinophilic infiltrations admixed with CD3+/CD4 + helper T lymphocytes, macrophages, and adjacent cardiomyocyte injury (Figures 3A, B, D–F). Immunostaining for major basic proteins demonstrated extensive staining, predominantly eosinophils in the endocardium (Figure 3C), with macrophage polarization toward the M2 phenotype (Figures 3G,H). These findings confirmed a definitive diagnosis of acute eosinophilic myocarditis (AEM), which was treated with intravenous methylprednisolone (1 g/day for 3 days), followed by oral prednisolone (1 mg/kg/day) with gradual dose tapering. Thereafter, the patient remained hemodynamically stable. The IABP was weaned off on day 12, and the patient was transferred from the ICU to the general ward.

**Figure 4:**
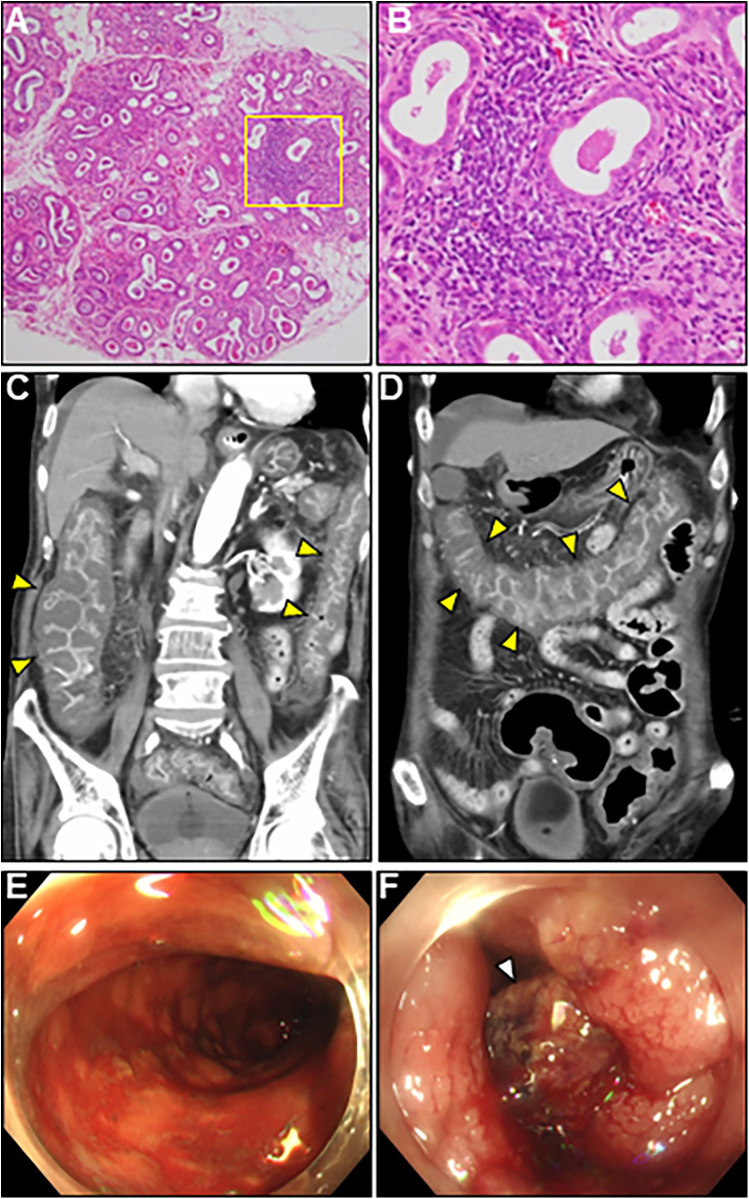
Lip gland histopathology, computed tomography (CT) and colonoscopy findings. **(A)** Pathology of a minor salivary gland biopsy (20× magnification). **(B)** Magnified view of the box in **(A)** at 40×. Hematoxylin and eosin staining showing focal lymphocytic sialadenitis with periductular aggregates of >50 lymphocytes. **(C,D)** Coronal enhanced CT revealing whole and homogeneous large bowel wall thickening characterized by submucosal edema and mucosal thickening with the disappearance of the haustral folds (arrowheads). Note the intact small bowel. **(E,F)** Colonoscopy revealed continuous diffuse mucosal inflammation from the rectum to the transverse colon. There are no vascular patterns, multiple ulcers with friability, mucosal desquamation (arrowhead), or contact bleeding.

A comprehensive diagnostic evaluation of eosinophilia, including eosinophilic granulomatosis with polyangiitis, hypereosinophilic syndrome, parasitic infections, and hematological malignancies, revealed negative results. Viral screening using paired serology for virus antibody titers was unremarkable (Supplementary Table S1). In addition, a workup for the unexplained eosinophilia was performed. Most importantly, a renewed interview with the patient's family revealed that the patient complained of oral dryness for >6 months and frequently drank too much liquid. We performed serologic testing for underlying systemic autoimmunity using stored blood samples obtained on admission. Strikingly, serologic testing using pre-corticosteroid treatment sera on admission demonstrated strong positivity for anti-SS-A and anti-SS-B antibodies (469 and 199 U/ml, respectively), strongly suggesting SS. Elevated levels of serum rheumatoid factor (31 IU/ml, normal: < 15 IU/ml), interleukin (IL)-4 (36.5 pg/ml, normal: < 3.9 pg/ml), IL-5 (50.9 pg/ml, normal: < 3.9 pg/ml), and antinuclear antibody titer (1:320) were also noted. Brain CT revealed fatty changes in the bilateral parotid glands. An abnormal Schirmer's test result and lip biopsy of the patient revealed focal lymphocytic sialadenitis with a focus score of ≥1 focus/4 mm^2^, meeting the 2016 ACR/EULAR classification criteria for pSS (4) (Figures 4A,B). Based on the above, the final diagnosis of AEM associated with pSS was established. On day 22, the patient showed an evident improvement in cardiac functional and structural abnormalities, as well as electrocardiographic findings, except for an LV apical aneurysm (Figures 2G,H; Supplementary Video S3, and S4). A follow-up brain MRI demonstrated complete recovery of AOM. Thereafter, the patient received a maintenance dose of prednisolone (20 mg/day) and underwent cardiac rehabilitation. The post-treatment course of the patient was complicated by digestive involvement (DI) including esophageal candidiasis and chronic atrophic gastritis. Accordingly, oral miconazole and a reduced dose of prednisone (10 mg/day) were prescribed. On day 51, the patient experienced three episodes of bloody stools, and the anticoagulant was discontinued. On day 52, the patient experienced hemorrhagic shock due to lower gastrointestinal bleeding with progressively impaired consciousness, necessitating tracheal intubation, catecholamine administration, frequent blood transfusions, and careful monitoring in the ICU. Enhanced CT showed diffuse, severe, and edematous thickening of the entire colonic wall despite intact mesenteric arterial circulation (Figures 4C,D). Colonoscopy revealed diffuse edematous mucosa, complete loss of vascular pattern, mucosal friability, and multiple superficial ulcers with contact bleeding in the rectum and sigmoid colon (Figures 4E,F). These lesions were observed in continuity with the rectum. Given the patient's underlying condition of pSS, CT and colonoscopy findings suggestive of ulcerative colitis (UC), the rapidly progressive course, and steroid resistance, fulminant UC was strongly suspected. However, we could not perform colon biopsies due to the rapid deterioration of the patient's condition. Despite intensive therapy, the patient eventually succumbed to septic shock on day 59. An autopsy of the patient was not performed because of the refusal by family members. We present a summarized illustration of the case presentation in Supplementary Figure S1.

## 3 Discussion

Herein, we describe a case of severe pSS presenting with AEM, which led to fatal systemic sequelae despite corticosteroid treatment. Our case offers the following four clinical lessons.

First, to the best of our knowledge, this is the first case of biopsy-proven AEM associated with pSS. The occurrence of AEM concomitant with the clinical features of pSS and the exclusion of other etiologies of eosinophilia established the final diagnosis of pSS-associated AEM. EMBs in all the previous case reports with pSS-associated myocarditis have revealed lymphocytic infiltration with predominant immunostaining for CD3+/CD8 + cytotoxic T lymphocytes, suggestive of an autoimmune response (5, 6). However, EMB in our case revealed marked eosinophilic and lymphocytic infiltration, with predominant immunostaining for CD3+/CD4 + helper T lymphocytes, suggestive of an allergic response. Additionally, circulating levels of IL-4 and IL-5 were highly elevated in our case. This case highlights a distinct subtype of pSS-associated myocarditis with a predominance of Th2-related cytokines. However, the exact mechanism by which Th2-related cytokines contribute to AEM remains unclear. A basic study using mouse models of experimental autoimmune myocarditis suggests that the eotaxin-CCR3 pathway is required for the localization of eosinophils to the myocardium (7). Therefore, the possible mechanism underlying Th2-related cytokine-mediated AEM in our case is postulated as follows: (i) unidentified upstream signals hyperactivate the Th2-related cytokines signaling. (ii) IL-5 induces systemic eosinophil production, accumulation, and activation and promotes their release from the bone marrow into peripheral circulation (8). (iii) Simultaneously, IL-4 induces the expression of eotaxin ligand in cardiac fibroblasts and macrophages. (iv) the eotaxin-CCR3 pathway efficiently recruit eosinophils expressing the CCR3 protein into the cardiac tissue. (v) Subsequent eosinophilic infiltration causes AEM. Further elucidation of the crosstalk between pSS and Th2-related cytokines signaling is desired.

Second, in the present case, AEM manifested before peripheral eosinophilia, leading to the delayed definitive diagnosis. Some previous reports have noted that peripheral eosinophilia may not occur in the early disease stage (9–11). Another research using large clinical databases confirmed that approximately one-fourth of cases with histologically-proven AEM did not involve eosinophilia (12). Interestingly, peripheral eosinophilia was only mild and transient, yet the EMB revealed profound eosinophilic infiltration in our case. Possible mechanism underlying this paradoxical eosinopenia remains unclear. However, in acute disease states, eosinophils may selectively accumulate in the myocardium (13). In contrast, bone-marrow eosinophil production cannot respond immediately. Later, the increased number of eosinophils is released from the bone marrow into the peripheral circulation. This phenomenon might explain such a transient paradoxical eosinopenia. Hence, tracking serial eosinophil counts is required.

AEM is rare, yet potentially fatal condition if left untreated. Eosinophils may activate cardiac mast cells, release cytotoxic granule proteins on cardiomyocytes, and play a major pathological role in AEM (14). Management of AEM consists of treating heart failure, preventing eosinophil myocardial infiltration, suppressing inflammation, and preventing thrombosis. Corticosteroids are the mainstay of AEM treatment, and patients in the early disease stage generally respond well to corticosteroids. For patients with progressive, refractory, or recurrent disease, the concomitant use of other immunosuppressants or intravenous immunoglobulin should be considered. Mepolizumab, an anti–IL-5 monoclonal antibody, may be another option to target eosinophil infiltration (15). However, in cases of advanced heart failure, non-pharmacological interventions including mechanical circulatory assist devices or heart transplantation, may be necessary (16). In our case, systemic and oral corticosteroids almost restored cardiac structure and function except for cardiac aneurysm, and the patient maintained the preserved cardiac function during his hospital course. Hence, our case highlights the crucial role of early immunosuppressive intervention in AEM.

Third, in our case, the clinical symptoms, imaging features, and good treatment response to corticosteroids eventually led to the diagnosis of AOM secondary to pSS. Although infections or drugs may cause hearing loss, the temporal relationship between the onset of hearing loss and potential ototoxic drug use, and the test results including paired virology and blood cultures ruled out the possibility of any conditions other than pSS.

Although hearing loss could be an under-recognized manifestation of SS, a systematic review and meta-analysis indicated a relatively high prevalence of hearing loss in patients with pSS exceeding 50% (17). Another large-scale observational study revealed a strong association between SS and chronic otitis media (18). Possible mechanisms of hearing loss in patients with SS include ototoxic medications, neural involvement, middle ear obstruction due to inflammatory secretions, and inflammation or dryness of mucous membranes in the eustachian tube. This case report highlights the importance of considering pSS in the differential diagnosis of hearing loss.

Fourth, the current case exhibited severe DI, including esophageal candidiasis, chronic atrophic gastritis, and fulminant UC. An association between inflammatory bowel disease and pSS has been documented in several reported cases (19–22). A randomized multicenter cohort study involving 438 patients with pSS revealed a DI prevalence of 16.2% (23), with chronic atrophic gastritis being the most frequent (30%), followed by primary cholestatic cholangitis (25%), autoimmune hepatitis (21%), altered esophageal motility (17%), and lymphocytic colitis (3%). Patients with pSS generally have a good prognosis; however, patients of pSS with systemic diseases have a poor prognosis. A GEAS-SS multicenter registry demonstrated that 13% of patients with pSS exhibited severe systemic disease requiring more intensive therapeutic management, with a mortality rate of 20%–36%.

In our case, gastrointestinal complications were considered most likely associated with severe systemic pSS. Because we could not perform gastrointestinal pathology, the possibility of immunosuppressant- or antibiotic-associated colitis (e.g., *cytomegalovirus*, *clostridium difficile*) could not be ruled out. Our patient's overall condition was very poor, and there were no room for alternative therapies including conventional immunosuppressive drugs and other biologic agents, including Rituximab or Abatacept. EMB after corticosteroid treatment and early comprehensive surveillance of systemic organs, including gastrointestinal pathology, might have provided useful information for predicting rapid deterioration.

Finally, several cases of pSS with other eosinophilic organ involvement (lungs or gastrointestinal tract) have reported (24, 25), suggesting that eosinophilic involvement may occur in various organs in patients with pSS. Clinicians should consider coexistent eosinophilic organ involvement and perform biopsy of affected organ tissues in patients of pSS presenting with any organ involvement accompanied by unexplained eosinophilia.

## 4 Conclusions

To the best of our knowledge, this is the first report of severe pSS complicated by AEM.

Despite considerable improvement in AEM following corticosteroid treatment, the patient developed severe systemic complications, including gastrointestinal events, ultimately leading to death. Clinicians should promptly perform an EMB to diagnose AEM in order to initiate early immunosuppressive intervention. Furthermore, in patients with pSS, prompt initiation of comprehensive monitoring for systemic organ involvement is required.

## Data Availability

The original contributions presented in the study are included in the article/Supplementary Material, further inquiries can be directed to the corresponding author.

## Supplementary material

Supplementary Table S1 Serological tests for viral infections.

Supplementary Figure S1 Timeline of the diagnostics, therapeutic interventions, and disease status of the current case. AEC, absolute eosinophilic count; AEM, acute eosinophilic myocarditis; Af, atrial fibrillation; AOM, acute otitis media; BP, blood pressure; BT, body temperature; cTnI, cardiac troponin I; CT, computed tomography; CRP, C-reactive protein; Dx, diagnosis; ECG, electrocardiography; EF, ejection fraction; EMB, endomyocardial biopsy; HR, heart rate; IABP, intra-aortic balloon pump; ICU, intensive care unit; IL, interleukin; IVD, intra-venous drip infusion; LV, left ventricular; LVD, left ventricular dysfunction; LVH, left ventricular hypertrophy; mPSL, methylprednisolone; MR, mitral regurgitation; MRI, magnetic resonance imaging; NT-proBNP, N-terminal pro-brain natriuretic peptide; PSL, prednisolone; pSS, primary Sjögren's syndrome; RF, rheumatoid factor; TTE, transthoracic echocardiography; UTI, urinary tract infection; WBC, white blood cell.

Supplementary Video S1 Pre-treatment transthoracic echocardiography: apical two-chamber view.

Supplementary Video S2 Pre-treatment color Doppler transthoracic echocardiography: apical four-chamber view.

Supplementary Video S3 Post-steroid treatment transthoracic echocardiography: apical two-chamber view.

Supplementary Video S4 Post-steroid treatment color Doppler transthoracic echocardiography: apical four-chamber view.
