# Supplementary material for: Biopsy-proven acute eosinophilic myocarditis as the initial manifestation of severe primary Sjögren's syndrome: a case report
Source: Front Cardiovasc Med. 2025 Oct 8;12:1683444. doi: 10.3389/fcvm.2025.1683444 (PMC12541781; doi:10.3389/fcvm.2025.1683444)
Supplement: Supplementary Table S1 — Serological tests for viral infections. [file Table1.docx]

| **Supplementary Table S1. Serological tests for viral infections** | | | | |  |  |  |  |  |  |
| --- | --- | --- | --- | --- | --- | --- | --- | --- | --- | --- |
| Virus | Type | Method | Day1 | Day 14 | Virus | Type | Method | Day1 | Day 14 |  |
| Adenovirus | 1 | NT | - | <4 | Echovirus | 1 | NT | - | <4 |  |
|  | 2 | NT | - | <4 |  | 3 | NT | - | <4 |  |
|  | 3 | NT | - | <4 |  | 4 | NT | - | 4 |  |
|  | 4 | NT | - | <4 |  | 5 | NT | 64 | 64 |  |
|  | 5 | NT | - | <4 |  | 6 | NT | - | <4 |  |
|  | 6 | NT | 32 | 16 |  | 7 | NT | - | 4 |  |
|  | 7 | NT | - | 4 |  | 9 | NT | - | <4 |  |
|  | 8 | NT | - | <4 |  | 11 | NT | 32 | 16 |  |
|  | 11 | NT | - | <4 |  | 12 | NT | - | <4 |  |
|  | 19 | NT | - | <4 |  | 13 | NT | - | 4 |  |
|  | 21 | NT | - | <4 |  | 14 | NT | - | 4 |  |
|  | 37 | NT | - | <4 |  | 16 | NT | - | <4 |  |
| Coxsackievirus | A2 | NT | 16 | 16 |  | 17 | NT | - | <4 |  |
|  | A3 | NT | - | 4 |  | 18 | NT | - | <4 |  |
|  | A4 | NT | - | <4 |  | 19 | NT | - | 4 |  |
|  | A5 | NT | - | <4 |  | 21 | NT | - | <4 |  |
|  | A6 | NT | - | 8 |  | 22 | NT | - | 8 |  |
|  | A7 | NT | - | 8 |  | 24 | NT | - | <4 |  |
|  | A9 | NT | - | <4 |  | 25 | NT | - | 4 |  |
|  | A10 | NT | - | <4 |  | 30 | NT | - | <4 |  |
|  | A16 | NT | - | <4 | HSV | 1 | NT | - | <4 |  |
|  | B1 | NT | - | <4 |  | 2 | NT | - | <4 |  |
|  | B2 | NT | 16 | 32 | Parvovirus | B19 | EIA | IgM, 0.25 | IgM, 0.25 |  |
|  | B3 | NT | - | 4 | HBsAg |  | CLEIA | Negative | - |  |
|  | B4 | NT | - | 8 | anti-HCV |  | CLEIA | Negative | - |  |
|  | B5 | NT | - | 4 | SARS-CoV-2 |  | RT-PCR | Negative | - |  |
|  | B6 | NT | - | <4 |  |  |  |  |  |  |
| CLEIA, chemiluminescent enzyme immunoassay; EIA, enzyme immunoassay; HBsAg, hepatitis B virus-specific antigen; HCV, hepatitis C virus; HSV, herpes simplex virus; NT, neutralization; RT-PCR, reverse-transcription polymerase chain reaction; SARS-CoV-2, severe acute respiratory syndrome coronavirus 2 | | | | | | | | | |  |
|  |  |  |  |  |  |  |  |  |  |  |
|  |  |  |  |  |  |  |  |  |  |  |
|  |  |  |  |  |  |  |  |  |  |  |
