# Supplementary figures and images for: Biopsy-proven acute eosinophilic myocarditis as the initial manifestation of severe primary Sjögren's syndrome: a case report
Source: Front Cardiovasc Med. 2025 Oct 8;12:1683444. doi: 10.3389/fcvm.2025.1683444 (PMC12541781; doi:10.3389/fcvm.2025.1683444)

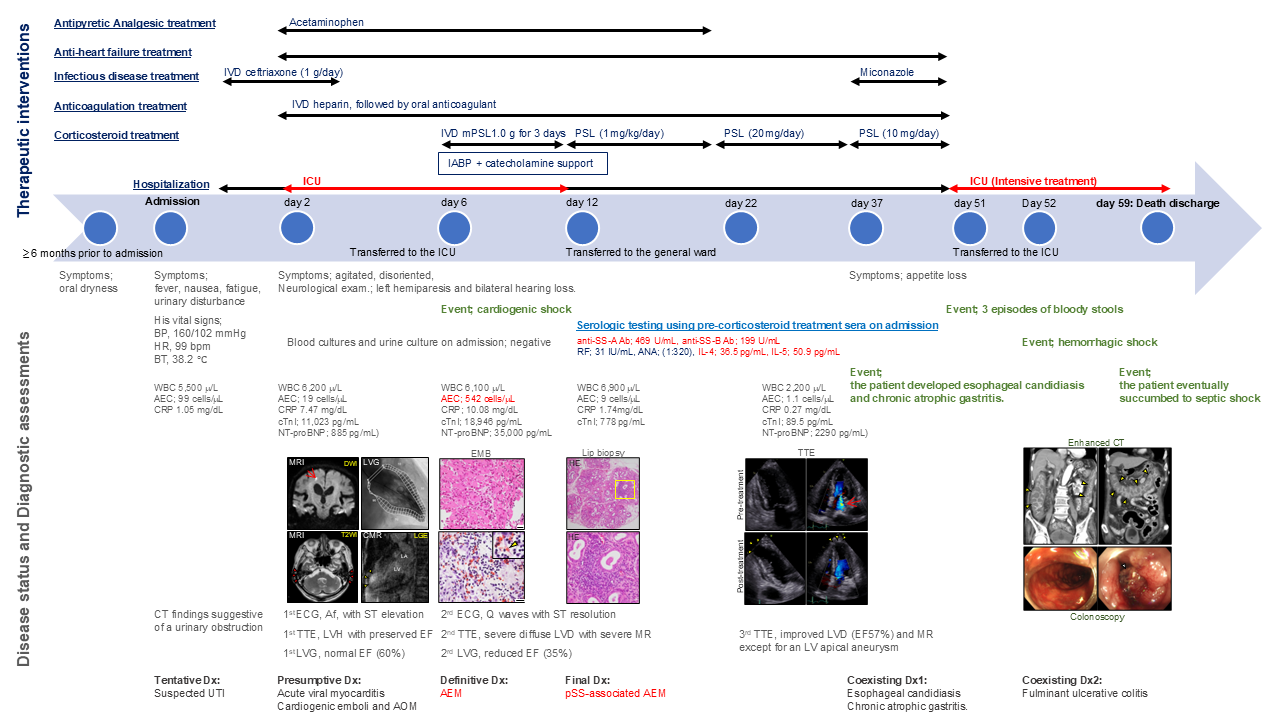

Supplement: Supplementary Figure S1 — Timeline of the diagnostics, therapeutic interventions, and disease status of the current case. AEC, absolute eosinophilic count; AEM, acute eosinophilic myocarditis; Af, atrial fibrillation; AOM, acute otitis media; BP, blood pressure; BT, body temperature; cTnI, cardiac troponin I; CT, computed tomography; CRP, C-reactive protein; Dx, diagnosis; ECG, electrocardiography; EF, ejection fraction; EMB, endomyocardial biopsy; HR, heart rate; IABP, intra-aortic balloon pump; ICU, intensive care unit; IL, interleukin; IVD, intra-venous drip infusion; LV, left ventricular; LVD, left ventricular dysfunction; LVH, left ventricular hypertrophy; mPSL, methylprednisolone; MR, mitral regurgitation; MRI, magnetic resonance imaging; NT-proBNP, N-terminal pro-brain natriuretic peptide; PSL, prednisolone; pSS, primary Sjögren's syndrome; RF, rheumatoid factor; TTE, transthoracic echocardiography; UTI, urinary tract infection; WBC, white blood cell. [file Image1.tif]
